# *Trichoderma* Species from Semiarid Regions and Their Antagonism Against the Microorganisms That Cause Pepper Wilt

**DOI:** 10.3390/jof11030174

**Published:** 2025-02-21

**Authors:** Erika Sireni Rodríguez-Martínez, Claudio Rios-Velasco, David Roberto Sepúlveda-Ahumada, José Juan Buenrostro-Figueroa, Kamila C. Correia, César Guigón-López, Mónica Alvarado-González

**Affiliations:** 1 Coordinación de Tecnología de Productos Hortofrutícolas y Lácteos, Centro de Investigación en Alimentación y Desarrollo, Cd. Delicias 33089, Chihuahua, Mexico; erodriguez222@estudiantes.ciad.mx (E.S.R.-M.); jose.buenrostro@ciad.mx (J.J.B.-F.); 2 Coordinación de Tecnología de Alimentos de la Zona Templada, Centro de Investigación en Alimentación y Desarrollo, A.C., Avenida Río Conchos s/n, Parque Industrial, Cd. Cuauhtémoc 31570, Chihuahua, Mexico; claudio.rios@ciad.mx (C.R.-V.); dsepulveda@ciad.mx (D.R.S.-A.); 3 Centro de Ciências Agrárias e da Biodiversidade, Universidade Federal do Cariri, Crato 63133-610, CE, Brazil; kamila.correia@ufca.edu.br; 4 Facultad de Ciencias Agrícolas y Forestales, Universidad Autónoma de Chihuahua, Km. 2.5 Carretera a Rosales, Poniente, Delicias 33000, Chihuahua, Mexico

**Keywords:** *Capsicum annuum*, antifungal activity, native *Trichoderma*, pepper wilt

## Abstract

Chili wilt is a significant challenge in producing jalapeño peppers, which has led to the implementation of strategies to help counteract or combat the microorganisms responsible for it. One of these strategies is the use of biological control microorganisms, such as *Trichoderma*, a fungus recognized as a natural enemy of the microorganisms that cause chili wilt. Therefore, this study aimed to isolate and identify *Trichoderma* species from the soils and roots of different plants, and evaluate their antagonism against *Rhizoctonia solani*, *Phytophthora capsici*, and *Fusarium* sp. Due to the complexity in identifying *Trichoderma* at the species level, performing a multilocus phylogenetic analysis was necessary, using the ITS, RPB2, and TEF1 regions. The species isolated were *T. afroharzianum*, *T. lentiforme*, *T. rifaii*, *T. brevicompactum*, *T. arundinaceum*, and *T. longibrachiatum*. Subsequently, they were used in three antagonism tests (dual culture, non-volatile organic compounds, and volatile organic compounds) against the phytopathogenic microorganisms. The tests demonstrated that the *Trichoderma* isolates could inhibit the mycelial growth of all three tested pathogens, obtaining the best results with the strains *T. brevicompactum* (19RCS), *T. lentiforme* (63DPS), *T. longibrachiatum* (71JES), *T. rifaii* (77JCR), and *T. afroharzianum* (24RQS, 87CCS, 88CCS and 17RCS). The strain with the best results in all three tests was 17RCS.

## 1. Introduction

Chili wilt disease is one of the main causes of production and economic losses in this crop. It is caused by several microorganisms, including *Phytophthora capsici*, *Rhizoctonia solani*, and *Fusarium* spp., which can act individually or synergistically [1]. These phytopathogens can damage all parts of the plant in the root, causing rotting and strangulation of the base of the stem. Therefore, the plant turns yellow, causing leaf fall, and when the disease is severe, it causes the death of the plant [2]. In countries such as China, India, and Mexico, this disease can lead to great economic losses for farmers. If conditions are favorable for the development of microorganisms, they can cause losses of up to 90% [3].

Currently, these pathogens are controlled using chemical fungicides. Unfortunately, the use of agrochemicals can lead to the development of resistance to fungicides in microorganisms. They also negatively impact the environment, as they contaminate the soil and aquifers, and their residues can be harmful to human health [4]. Given the negative environmental impact and the problems associated with the excessive use of fungicides to suppress fungal diseases, ecofriendly alternatives have been sought, such as biological control, which consists of using microorganisms that naturally control phytopathogens [5].

The use of biological control agents has several benefits for agriculture. One of them is that it uses microorganisms that are natural enemies of pathogens, promote plant growth or induce plant resistance mechanisms to biotic or abiotic stress, and also have no or minimal environmental impact compared to that of chemical control [6].

*Trichoderma* fungus has been used for biological control due to its multiple mechanisms of action for the suppression of pathogens, such as competition for space, mycoparasitism, and antibiosis, among others [5]. Their combination depends on the species and allows better control of phytopathogenic fungi.

Competition for space and nutrients against other soil microorganisms is the first mechanism of action of *Trichoderma* since there is limited availability of nutrients [7]. *Trichoderma* is attracted by plant exudates, rich in carbohydrates, amino acids, organic acids, and vitamins, among others, so it is located directly in the rhizosphere on the roots, or seeks to enter them [8]. Faster colonization of the available space requires the participation of enzymes and secondary metabolites [9].

Mycoparasitism occurs during the antagonistic interaction between *Trichoderma* and the pathogenic microorganism, where the former benefits from the latter. This consists of the following events: recognition of the prey microorganism; attack, penetration, and death of the pathogen. *Trichoderma* recognizes the pathogen due to diffusible signals such as the segregation of chitinases that, when detected, begin to produce cell wall-degrading enzymes and direct the growth of its hyphae towards the pathogenic fungus. This phenomenon is known as chemotrophic growth [10]. In addition, different species can secrete a wide variety of secondary metabolites that may contribute to their mycoparasitism and antibiotic action [11]. Most *Trichoderma* species are capable of producing volatile and non-volatile compounds such as acetaldehyde, ethylene, acetone, and carbon dioxide, and some produce antibiotics such as trichodermin, viridin, and ergokonin, responsible for their anti-phytopathogenic properties [12].

The metabolites produced by *Trichoderma* enable it to thrive in diverse ecosystems and adapt to different environmental conditions. For this reason, many researchers believe it is essential to isolate and study species from extreme ecosystems, such as arid and semi-arid soils [13]. Additionally, there is a significant benefit in utilizing native strains already adapted to the environment relevant to the crop of interest. This finding has been demonstrated by Guigón-López et al. [14], who isolated highly effective strains from various locations in Chihuahua for controlling *P. capsici*.

The mechanisms used by *Trichoderma* to control different pathogens may vary depending on *Trichoderma* species and strains and the target pathogen [15]. Because of this, selection of *Trichoderma* isolates for biocontrol of different pathogens usually characterizes their biocontrol mechanisms and defines those with the highest antagonistic activity [2]. Therefore, the aim of this study was (1) to morphologically and molecularly identify *Trichoderma* isolated from the soil and roots of jalapeño chilli pepper crops and wild plants; (2) to test their antagonism against the microorganisms causing chili wilt; and (3) to select the species with the most significant potential to be used in biological control of chili wilt disease.

## 2. Materials and Methods

### 2.1. Sampling

In northern Mexico, in the south-central zone of the state of Chihuahua, soil and root samples were collected from jalapeño chili plants established in agricultural areas with a semi-arid climate. Two plots were selected in each of the following municipalities: Camargo (27°38′28.03′ N, 105°13′30.54′ E and 27°38′27.59′ N, 105°13′27.19′ E), Delicias (28°8′22.68′ N, 105°33′19.56′ E and 28°12′51.02′ N, 105°24′9.27′ E), Jiménez (27°1′1.06′ N, 105°0′13.47′ E and 27°1′2.54′ N, 105°0′16.54′ E), Meoqui (28°20′40.32′ N, 105°33′1.98′ E and 28°23′19.53′ N, 105°33′4.33′ E), Rosales (28°11′0.74′ N, 105°32′23.46′ E and 28°11′57.90′ N, 105°33′44.81′ E), and Saucillo (27°59′20.29′ N, 105°17′28.99′ E and 27°59′25.12′ N, 105°17′7.76′ E). The primary condition for selecting plots was that no *Trichoderma*-based products had been applied during the crop cycle or in previous years.

In each plot, a zigzag pattern was used to collect the samples. From each jalapeño chili pepper plot, ten healthy plants, roots, and one kg of soil from the rhizosphere were collected randomly. Additionally, adjacent to the jalapeño chili pepper plot was a wild plant area, with random collection of samples of roots and soil from the rhizosphere. All samples were stored at 4 °C in the laboratory until processing.

### 2.2. Isolation and Morphological Identification of Trichoderma spp.

Soil samples were processed using the serial dilution method in sterile distilled water until the dilution reached 10^−5^. A 0.5 mL aliquot of each dilution was taken and inoculated onto a Petri dish containing rose Bengal selective agar (MgSO_4_.7H_2_O, 0.2 g; K_2_HPO_4_, 0.9 g; KCl, 0.15 g; NH_4_NO_3_, 3.0 g; glucose, 3.0 g; agar, 15 g; rose Bengal, 0.15 g; chloramphenicol, 0.25 g; captan, 0.04 g; pH 6.5) [16]. The plates were then incubated at 27 °C until fungal colony development was observed.

Root processing began with the separation of the roots based on their diameter. Then, roots were cut into 1 cm fragments. These fragments were immersed in a 2% sodium hypochlorite solution for 30 s, rinsed in sterile distilled water, placed on sterile filter paper, and allowed to dry at ambient temperature. Subsequently, they were transferred to Petri dishes containing rose Bengal agar. The plates were incubated at 27 °C for 48 to 120 h until colony development was observed.

Morphological identification was conducted using mounts on slides, fixed with lactoglycerol. The slides were examined under a compound microscope (Motic, Richmond, BC, Canada) to examine the microscopic structures and identify the isolates exhibiting typical characteristics of the *Trichoderma* genus. These characteristics include hyaline conidiophores that are branched but not verticillate, individual or in groups, hyaline conidia of one cell, ovoid in shape, and born in small clusters [17]. A total of 88 isolates were obtained from soils and roots. The isolates obtained were stored in 50 mL conical tubes (Corning™, Corning, NY, USA) containing sterile distilled water at 4 °C until further use for a stock solution of conidia and in a culture medium containing glycerol at −80 °C.

### 2.3. Molecular Identification

DNA extraction was performed for isolates selected for their outstanding antagonistic properties. Mycelium from each selected *Trichoderma* from 3 days of incubation in PDA at 27 °C was processed using the Quick-DNA Fungal/Bacterial Miniprep Kit™ from Zymo Research (Irvine, CA, USA) as per the manufacturer’s instructions. The extracted DNA was quantified using a Thermo Scientfic™ Nanodrop 2000c (Thermo Fisher Scientific™, Waltham, MA, USA) and visualized by performing agarose gel electrophoresis at 0.8%, separating the DNA bands at 80 volts for 1 h. DNA was stained with Diamond Nucleic Acid Dye™ (Promega Corporation, Madison, WI, USA).

For the polymerase chain reaction (PCR) of *Trichoderma* isolates, three primer sets were used: ITS1F (TCTGTAGGTGAACCTGCGG) and ITS2R (GCTGCGTTCTTCATCGATGC) for ITS [18,19]; EF1728F (CATCGAGAAGTTCGAGAAGG) and EF1986R (TACTTGAAGGAACCCTTACC) for EF [19,20]; and RPB2F (GAYGAYMGWGATCAYTTYGG) and RPB2R (CCCATRGCTTGTYYRCCCAT) for rpb2 [19,21]. Each PCR sample tube contained 12.5 µL of PCR master mix (Promega, Corp.), 2.5 µL of each primer, 2 µL of DNA, and 5.5 µL PCR-grade water. Amplifications were performed using a BioRad model C1000 thermal cycler (Bio-Rad, Hercules, CA, USA) under the following conditions: initial denaturation at 94 °C for 4 min; then, 35 cycles of denaturalization at 94 °C for 30 s, annealing at 55 °C for 30 s, and extension at 72 °C for 1 min; concluding with an extension at 72 °C for 10 min. The quality of the PCR products was verified by agarose gel electrophoresis. For ITS primers, a 1.2% agarose gel was run at 80 volts for 90 min. For the EF1 primers, a 2% agarose gel was used with a running voltage of 100 for 60 min and a 1:4 dilution of the PCR products was necessary. A 2% agarose gel was used with a voltage of 100 for 90 min for the RPB2 primers. The PCR products from *Trichoderma* isolates and fungal *R. solani* and *Fusarium* isolates were sequenced at Macrogen^®^ (Seoul, Republic of Korea).

### 2.4. Phylogenetic Analysis

Forward and reverse sequences were assembled using the Staden Package [22]. The sequences generated in the current study were deposited in GenBank (Table 1).

Multiple sequence alignments for each locus were independently performed using MEGA 7.0.14 [23], and adjustments were manually performed where necessary. The alignment of each locus was loaded in SequenceMatrix v.1.8 [24] to build the concatenated matrix.

The phylogeny for each locus (TEF, ITS, and RPB2) and the concatenated matrix were inferred under the maximum likelihood (ML) criterion. The ML analyses were performed in a RAxML-HPC2 [25] implemented on the CIPRES Science Gateway portal (https://www.phylo.org/portal2/home.action, accessed on 23 September 2023). ML tree searches were performed under the GTRGAMMA model with 1000 pseudoreplicates.

A maximum likelihood tree of the *Trichoderma* species was inferred from the combined dataset of the rDNA ITS regions and EF-1α and RPB2 genes. Bootstrap support (>70%) values for maximum likelihood and maximum parsimony are shown on the nodes. Isolates characterized in this study are highlighted in boldface.

### 2.5. Isolation and Identification of Phytopathogenic Fungi

Two phytopathogenic fungi were isolated from diseased jalapeño chili pepper plants. In the laboratory, roots and stems of diseased plants were separated and cut into approximately 1 cm fragments and disinfected with 2% sodium hypochlorite for 30 s, then rinsed with sterile distilled water and allowed to dry on sterile filter paper. Five fragments were placed in Petri dishes with PDA. The dishes were incubated at 27 °C. The colonies that developed in the dishes were then transferred to new Petri dishes with PDA to obtain pure cultures.

The fungi were identified based on their morphological characteristics, as well as the generation sequencing of the RPB2, ITS, and EF for *Fusarium* sp. and the ITS for *Rhizoctonia solani*.

The *P. capsici* strain used in this work was provided by Dra. Fernández-Pavía obtained from a collection belonging to the Institute of Agricultural Research and Foresters of the UMSNH. This isolate was previously identified by comparative morphology based on sexual and asexual characteristics by Reyes-Tena et al. [26].

### 2.6. Antagonism Tests In Vitro

#### 2.6.1. Dual-Culture Test

In the dual-culture test, the antagonistic abilities of *Trichoderma* isolates were evaluated against the phytopathogens *R. solani*, *Fusarium* sp., and *P. capsici.* In this setup, the two microorganisms coexist in the same space without barriers, allowing *Trichoderma* to employ various control mechanisms against target pathogens.

Posteriorly, a disc measuring 8 mm, with 3-day-old *Trichoderma* mycelia, was placed in a Petri dish containing PDA and the disc of phytopathogen mycelia on the opposite side at the same distance. The plates were then incubated at 27 °C for 5 days. The control consisted solely of a disc of the mycelial culture of the pathogen placed in a Petri dish with PDA. Each *Trichoderma* isolate was tested by confronting it with the three pathogens identified for evaluation. Daily measurements of the radial growth of the fungi in these confrontations were taken using a vernier caliper every 24 h, up to the moment of contact between the two fungi, and then the percentage of inhibition was calculated using the following formula:

$$\%INH=\frac{R1-R2}{R1}\times 100$$

where *R*1 corresponds to the radial growth of the control and *R*2 to the radial growth of the treatment [27].

#### 2.6.2. Volatile Organic Compound Activity

The activity of volatile organic compounds was determined by applying the methodology described by Baiyee et al. [16]. For this test, PDA discs with mycelial growth of the *Trichoderma* isolates and PDA agar discs with mycelial growth of the three pathogens were utilized. A disc of the *Trichoderma* isolate was placed in the center of a Petri dish containing PDA, while a disc of the pathogenic microorganism was placed in the center of another Petri dish with PDA; the lids of the dishes were then removed, allowing the two dishes to be confronted with the *Trichoderma* placed near the phytopathogen. Once confronted, the dishes were sealed together. This procedure was repeated for each *Trichoderma* isolate and each of the pathogens. As a control, a disc of the pathogenic fungus was placed in one Petri dish with PDA, while another Petri dish contained only PDA. The combination setups were incubated at 27 °C until the control completely covered the dish. The diameter of growth of the pathogenic fungus was measured every 24 h, and the percentage of growth inhibition was calculated by comparing the control against the treatment with the following formula:

$$\%INH=\frac{D1-D2}{D1}\times 100$$

where *D*1 corresponds to the growth diameter of the control and *D*2 to the growth diameter of the treatment [28].

#### 2.6.3. Non-Volatile Organic Compound Activity

The cellophane test described by Kredics et al. [9] was used for this study. A sterile cellophane membrane was placed in a Petri dish with PDA. In the central part, 8 mm disks of mycelial culture of each *Trichoderma* isolate were placed on it. The Petri dishes were incubated at 27 °C for 3 days. The cellophane was removed, ensuring no *Trichoderma* fragments remained on the medium. Then, an 8 mm disk with a mycelial growth of the phytopathogenic microorganism to be evaluated was placed in the center of the Petri dish, and incubation was continued at the same temperature until the control fungus completely covered the Petri dish. The control culture consisted of placing the disc with mycelial growth of the pathogenic fungus without prior inoculation of the *Trichoderma* isolate. Each *Trichoderma* isolate was evaluated by the three pathogens. The growth diameter of each treatment was measured daily. The percentage of growth inhibition was obtained by comparing the control against the treatment with the following formula:

$$\%INH=\frac{D1-D2}{D1}\times 100$$

where *D*1 corresponds to the growth diameter of the control and *D*2 to the growth diameter of the treatment [29].

A total of 88 *Trichoderma* isolates were initially confirmed through morphological analysis. Subsequently, three in vitro tests on antagonism were conducted, selecting 20 isolates based on their antagonistic abilities. Finally, the 20 selected isolates were molecularly identified. (Procedure described in the Supplementary Materials).

### 2.7. Experiment Design and Statistical Analysis

A completely randomized design was used in the three tests, with 3 replications. Each experimental unit corresponds to a Petri dish and the response variable corresponds to the percentage of mycelial growth inhibition of each phytopathogenic fungus. An analysis of variance and a Tukey’s test (*p* = 0.05) of separation of means were carried out, using the SAS OnDemand software.

## 3. Results

### 3.1. Isolation and Morphological Identification of Trichoderma spp.

**Figure 1:**
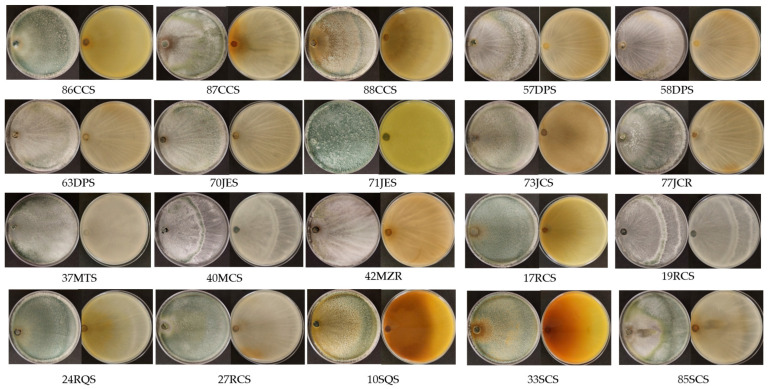
Growth of Trichoderma strains in Petri dishes (90 mm diameter) with PDA medium for 5 days of incubation.

A total of 88 *Trichoderma* isolates were collected. Based on morphological analysis and antifungal activity through dual-confrontation tests, 20 isolates that stood out for their antagonistic ability were selected for study in greater detail. A total of 9 isolates were obtained from jalapeño chili pepper plants (8 from the rhizosphere and 1 from the root) and 11 isolates from wild plants (10 from the rhizosphere and 1 from the root) (Table 1 and Figure 1).

### 3.2. Isolation and Morphological Identification of Phytopathogenic Microorganism

**Figure 2:**
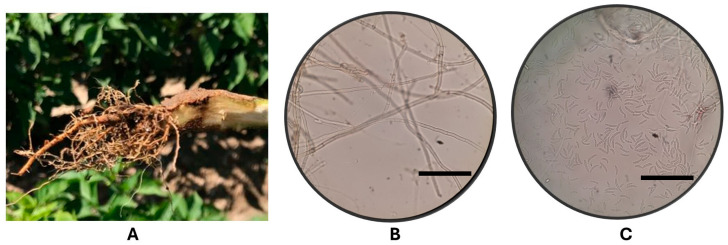
(**A**) Diseased root of jalapeño pepper plant. (**B**) *Rhizoctonia solani*. (**C**) *Fusarium* sp. Scale 45 µm (**B**,**C**).

The *R. solani* (RHCIAD) strain (PP512540 GenBank ascension number) and *Fusarium* sp. were isolated from diseased pepper (*Capsicum annum* L.) root in Jiménez, Chihuahua, Mexico. The *Phytophthora capsici* (PHC) strain CPV-283 (AR26) was isolated from diseased pepper (*Capsicum annum* L.) plants in Yurécuaro, Michoacán, Mexico. Registration of the Fusarium and Phytophthora strains in GenBank is currently in process (Figure 2).

### 3.3. Phylogenetic Analysis

**Figure 3:**
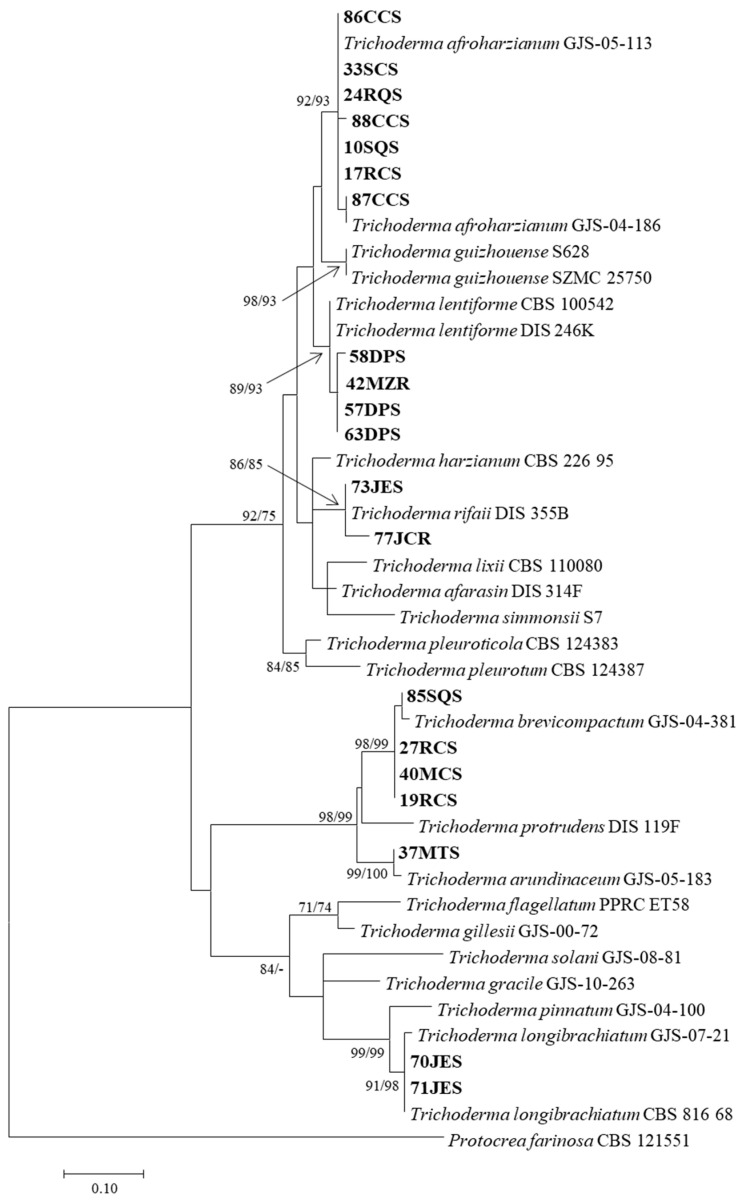
Maximum likelihood tree of the *Trichoderma* species inferred from the combined dataset of the rDNA ITS regions and EF-1α and RPB2 genes. Bootstrap support (>70%) values for maximum likelihood and maximum parsimony are shown on the nodes. *Trichoderma* isolates selected and characterized in this study are highlighted in boldface.

The three-gene phylogenetic analysis consisted of the 20 *Trichoderma* isolates selected in this study, as well as 23 reference sequences from the *Brevicompactum*, *Harzianum*, and *Longibrachiatum* clades. *Protocrea farinosa* (CBS 121551) was used as the out-group. A total of 2352 characters were analyzed: ITS = 684, TEF = 462, and RPB2 = 1206. Among the 20 *Trichoderma* isolates, 13 were identified to be in the *Harzianum* clade, 5 were identified to be in the *Brevicompactum*, and 2 isolates were identified to be in the *Longibrachiatum* clade based on analysis of the combined ITS, TEF, and RPB2 gene sequences (Figure 3).

The isolates obtained are grouped into six species of *Trichoderma*. In the harzianum clade, seven (35%) isolates were identified as *Trichoderma afroharzianum*, four isolates clustered with reference isolates of *Trichoderma lentiforme*, and two isolates clustered with reference isolates of *Trichoderma rifaii*. Of the five isolates belonging to the *Brevicompactum* clade, one isolate was identified to be *Trichoderma arundinaceum* and four isolates were identified with *Trichoderma brevicompactum*. The two isolated from the *Longibrachiatum* clade grouped with the type species of the clade, being identified as *Trichoderma longibrachiatum*. DNA sequence alignment data were deposited in GenBank (Table 1).

Among the seven *T. afroharzianum* isolates, five were collected from the rhizospheric soil of *C. annum* cultivation and two were obtained from the wild plant *Amaranthus* sp. Three of the four isolates of *T. lentiforme* were collected from rhizospheric soil of *Picea* sp. and one from *Sorghum halepense* (L.) *Pers* roots.

The two isolates of *T. rifaii* were collected from the root and soil of the *C. annum* cultivation. All *T. brevicompactum* isolates were obtained from *C. annum* soil. The *T. arundinaceaum* isolate was obtained from *Solanum elaeagnifolium Cav.* soil. And the *T. longibrachiatum* isolates were sourced from *S. kali* soil.

### 3.4. Evaluation of Antagonism of Trichoderma spp. Against Phytopathogenic Microorganisms

#### 3.4.1. Dual-Culture Test

**Figure 4:**
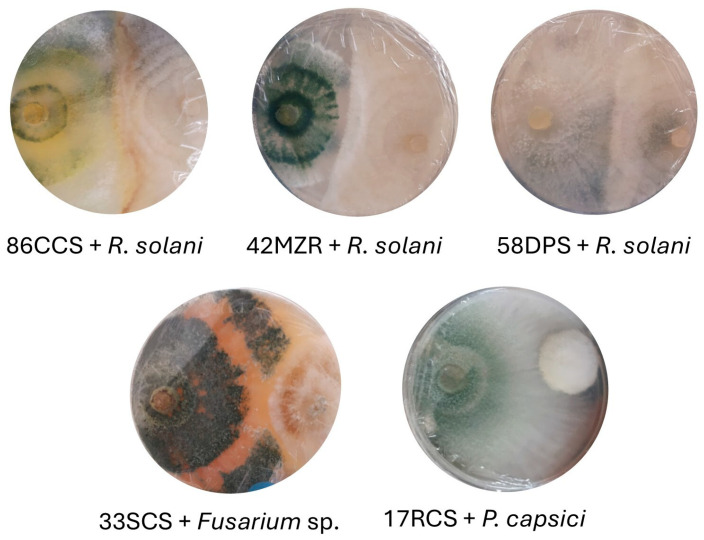
Growth of *Trichoderma* against phytopathogens in the dual-culture test in Petri dishes (90 mm diameter) with PDA medium for 5 days of incubation. It can be seen that *T. afroharzianum* 86CCS and *T. lentiform*, strains 42MZR and 58DPS, showed similar moderate inhibition on *R. solani*. Growth inhibition was more pronounced for *T. afroharzianum* 33SCS against *Fusarium* sp. and especially *T. afroharzianum* 17RCS against *P. capsici*. The inhibition means for each test are presented in Table 2.

All *Trichoderma* isolates demonstrated the ability to inhibit the growth of at least one plant; some examples are shown in Figure 4. Overall, their growth rates were generally faster than those of the pathogens. The percentage of growth inhibition observed was as follows: for *R. solani* it ranged from 18.69 to 65.37; for *P. capsici* from 29.14 to 81.72; and for *Fusarium* sp. from 24.30 to 68.98 (Table 2 and Table S2).

Twelve *Trichoderma* strains demonstrated a statistically significant superior percentage of inhibition against *R. solani*. For *P. capsici*, six strains showed significantly higher inhibition percentages (*p* ≤ 0.05). In the case of *Fusarium* sp., five strains were notable for their ability to inhibit the pathogen effectively (INH around 60%).

#### 3.4.2. Non-Volatile Organic Compound Activity

Non-volatile compounds of all the *Trichoderma* strains showed significant effects on the growth of the three fungal pathogens. In the case of *R. solani*, fifteen strains achieved a percentage inhibition of mycelial growth, with strain 57DPS being the one that obtained the highest value (60.27%). While, in *P. capsici*, six strains (17RCS, 19RCS, 33SCS, 37MTS, 40MTS, and 85SCS) were statistically superior, with strain 85SQS being the one that obtained the highest value (86.93%). On the other hand, in terms of inhibition of the growth of *Fusarium* sp., strain 73JES was the most effective, achieving a complete inhibition rate of 100%.

#### 3.4.3. Volatile Organic Compounds

The inhibitory activity of the VOCs from *Trichoderma* was lower than that of the non-volatile compounds; the VOCs of the *Trichoderma* strains did not achieve inhibition of the growth of *R. solani*, with values generally below 10%. In the case of *P. capsici*, 15 *Trichoderma* strains showed inhibition percentages that were statistically equal, while in *Fusarium* sp. this value was 18 isolates. In both, *P. capsici* and *Fusarium* sp., the inhibition effect began to manifest from the first 24 h and exceeded 50%.

## 4. Discussion

Soil-borne pathogenic microorganisms that cause chili wilt disease pose a significant challenge to jalapeño chili pepper production. To address this issue, several strategies have been sought to prevent and control this disease, such as integrating biological control organisms. In the present work, 89 isolates of *Trichoderma* were obtained from healthy chili bell pepper plants, of which 20 isolates were selected based on their taxonomic location and characterization of their antagonistic efficiency for the phytopathogens that affect the roots of chili pepper.

The interest in the isolation and the accurate identification of *Trichoderma* species has been increasing over the years [30]. It is currently known that the accurate identification of *Trichoderma* species is complex, so it has been sought to standardize the molecular identification process to achieve a more accurate identification at the species level. Several authors have established that the gene regions necessary to support a suitable identification are ITS, TEF1-α, and RPB2 [31,32]. Therefore, this study used datasets from all three regions to analyze the phylogenetic relationships among *Trichoderma* species.

The species identified belonged to three clades that are important, either because they are known biological control agents or species with great biotechnological potential.

The *Harzianum* clade includes several species of economic importance due to their use in biological control; something to highlight about these species is that they have been isolated in different substrates, geographic locations, and hosts [33]. In this work, three species belonging to this clade, *T. afroharzianum*, *T. lentiforme*, and *T. rifaii* were obtained.

*T. afroharzianum*

*T. afroharzianum* was found in rhizospheric soil of jalapeño chili pepper plants at the Rosales, Saucillo, and Camargo locations (17RCS, 33SCS, 86CCS, 87CCS, and 88CCS) and in the rhizospheric soil of wild *Amaranthus* sp. plants at the Saucillo and Rosales locations (10SQS and 24RQS). *T. afroharzianum* is reported to be common on different types of substrates, including soil, roots, and other fungi, and it is also widely distributed geographically [33]. For example, it has been reported in Algeria [34]; in soil of tomato plants in Egypt [35]; in roots of healthy wheat plants in Spain [36]; in soil of healthy tomato plants in China [37]; and in onion plants in Brazil [38], among other locations.

Strain *T. afroharzianum* 17RCS stood out against the three phytopathogens, presenting mycelial growth inhibition percentages higher than 50% in most of the tests, except for the activity of volatile compounds against *R. solani* (5.30%) and in the dual-culture test with *P. capsici* (48.95%). In contrast, and despite belonging to the same species, strain *T. afroharzianum* 10SQS was the worst performer, obtaining a value higher than 50% only in the dual-culture test against *P. capsici* (74.2%).

In general, this species has been already recognized for its good antagonistic activity against multiple phytopathogenic microorganisms such as *F. oxysporum*, *F. solani*, *Macrophomina phaseolina*, *Pythium ultimun*, [39], *Fusarium graminearum* [36], and *Alteraria alternata* [35].

In similar studies in which dual cultures of this species were analyzed against the pathogens evaluated here, very similar results were obtained, where the percentage of inhibition was around or slightly higher than 50% [40,41].

*T. lentiforme*

*T. lentiforme* was found both in rhizospheric soil of *Picea* (57DPS, 58DPS, and 63DPS) and in the root of *S. halepense* (42MZR), which coincides with the typical habitats of the species since it has generally been found in soil and acting as an endophyte. An interesting fact is that this species is generally found in tropical areas in America [30,33,42], although Jambhulkar et al. [43] reported the presence of this species in Rajasthan, India, in rhizospheric soil of soybean plants, and Yin et al. [44] even managed to isolate it from a fresh leaf of the mangrove *Bruguiera gymnorrhiza* collected from Dong Zhai Gang National Nature Reserve in Hainan Province, China. But in general, in areas very different from the environment in which it was detected in this work, characterized by low rainfall and extreme temperatures, there is a high contrast between day and night. This may reflect the broad ecological plasticity of *T. lentiforme*, which allows it to adapt to such contrasting environments.

All isolates of *T. lentiforme* species excelled in antagonism by non-volatile compounds against *R. solani*, while *P. capsici* and *Fusarium* sp. were superior in inhibition by VOCs. Although the antagonistic capacity due to these compounds has not been thoroughly investigated yet, some specific secondary metabolites, such as polyketides and peptaibols, have recently been studied for their potential antimicrobial activity [44,45].

*T. rifaii*

*T. rifaii* stands out within the *Harzianum* clade for having the smallest conidia. This fungus is commonly found as an endophyte, although it can sometimes be located in soil [33]. This is in agreement with what was reported in the present study since isolate 73JES came from rhizospheric soil of the *Salsola kali* plant, while the isolate 77JCR was found in the root of the *C. annuum* plant.

Regarding the inhibition capacity of the three pathogens, both strains were very similar against *P. capsici* and *Fusarium* sp. since they both stood out in the analysis of VOCs. Notably, strain 77JES inhibited 100% of the growth of *Fusarium* sp. using the non-volatile compounds. On the other hand, when confronted with *R. solani*, strain 77JCR was significantly superior in all three tests, while strain 73JES only stood out in the non-volatile compounds test.

The *Brevicompactum* clade includes the species *T. brevicompactum*, *T. arundinaceum*, *T. turrialbense*, and *T. protrudents*. Many species previously identified morphologically as *T. harzianum* have been reclassified to *T. brevicompactum* based on phylogenetic analyses of their ITS, RPB2, and TEF1α sequences. All species belonging to this clade can produce mycotoxins: trichodermin or harzianum A [46,47]. In the present study, only *T. brevicompactum* and *T. arundinaceum* species were found within this clade; they were detected in rhizospheric soil.

*T. brevicompactum*

*T. brevicompactum* was found on chili plants at the Rosales and Meoqui locations (19RCS, 27RCS, and 40MCS) and on *Amaranthus* sp. at Saucillo (85SQS).

*Brevicompactum* species have the property of growing at relatively high temperatures (30–32 °C). One distinctive morphological characteristic is a dense sporulation in compacted areas, which displays an olive green to gray-green coloration. In addition, it develops subglobose conidia [46].

*T. brevicompactum* has been reported in Algeria, where it showed potential to control *B. cinerea* through dual-culture and volatile compound activity analysis [48], In Paraguay, it was found in rhizosphere soil of *Solanum lycopersicum* [49]. In China, it was found in rhizosphere soil of Crocus sativus and, in addition, it proved to be efficient in controlling *F. oxysporum* through multiple mechanisms such as volatiles, non-volatiles, and fermented broth extract [50]. It was also reported in Indonesia, where they tested its effectiveness in inhibiting the growth of *R. solani*, obtaining inhibition results of up to 77%, which is superior to the results obtained in this study [51]. In the case of dual culture against *P. capsici*, results were also lower than those reported by other authors for this species [52]. While they reached inhibition percentages of up to 70%, in this study only 60% was achieved.

On the other hand, in the case of *Fusarium* sp. it is worth noting that higher inhibition percentages were obtained than those reported in other similar work. In that study, the inhibition was up to 52% in *Fusarium oxyxsporum*, while in our study up to 68% inhibition was reported.

*T. brevicompactum* is known to produce trichodermin, which is recognized for its antifungal capacity. This compound is a trichothecene; it affects other microorganisms by inhibiting protein synthesis by preventing the formation of peptide bonds in the peptidyl transferase center of the 60S ribosomal subunit [53].

*T. arundinaceum*

The *T. arundinaceum* species shows a strong morphological similarity to *T. brevicompactum*. However, phylogenetic analyses have demonstrated a considerable phylogenetic distance between them. Some strains previously classified as *T. harzianum* and *T. viride* have been reclassified as *T. arundinaceum*. It is important to note that both strains were previously reported to produce harzianum A [47]. This compound is a trichothecene that is not only phytotoxic but also presents antagonistic activity against phytopathogenic fungi that can induce plant genes related to the defense response [54,55].

In the present study, *T. arundinaceum* was obtained in rhizospheric soil of *S. elaeagnifolium* (37MTS); it has not been reported in Mexico; therefore, this would be the first report of *T. arundinaceum* in the country. In our study, this species stood out mainly in the antagonistic dual culture of *R*. *solani* and in non-volatile compounds against *P. capsici*. The results against *R. solani* were similar to those reported by Cardoza et al. [55], where they found three isolates *of T. arundinaceum* in rhizospheric soil of bean plants and tested their efficacy against this pathogenic fungus. They showed that although the strains produced harzianum A, all of their isolates showed an outstanding antifungal effect regardless. This may indicate the presence of other important metabolites in the antagonism of pathogenic fungi. Building on our findings, it is possible that these metabolites are found in the non-volatile compounds since the best results in this species were obtained in the direct contact tests.

The *Longibrachiatum* clade comprises at least 26 phylogenetic species, including *T. reesei* and *T. longibrachiatum* [56]. *T. longbrachiatum* was found at the Jimenez location in rhizospheric soil of *Salsola kali* (70JES and 71JES). The morphological characteristics of these isolates coincided with what is reported in the literature since they present very specific characteristics, such as yellow coloration on PDA medium, as well as the production of conidiophores in sparse aerial mycelium that accumulates in pustules of cottony appearance [57].

It is considered a cosmopolitan fungus, typically from tropical regions [57], although it has been reported in countries such as Algeria [34], Paraguay [49], and Pakistan, in soils with *Capsicum annuum* crops [58].

Both strains of *T. longibrachiatum* (70JES and 71JES) were antagonistic against *R. solani* by non-volatile compounds, while against *P. capsici* and *Fusarium* sp. the method that stood out most was inhibition by VOCs, with inhibition values ranging from 35 to 45%. These results are in agreement with those obtained by Sridharan et al. [59], in which they tested the antagonistic potential of *T. longibrachiatum* against *Sclerotium rolfsii* and *Macrophomina* phaseolina, both soil fungi that act as causal agents of various diseases in a wide variety of hosts. In that study, they also analyzed the VOC profile of the interaction of *T*. *longibrachiatum* with pathogens, where they found a total of 138 compounds, some of which were reported as antifungals. In addition, they noted changes in the VOC profile depending on the fungus with which it was interacting, indicating that *Trichoderma* modified the expression of its secondary metabolites depending on the phytopathogen with which it interacts. On the other hand, in the dual-culture study against *P. capsici*, the growth inhibition results were found to be much higher than those reported in studies such as that of De la Cruz-Quiroz [60], where they obtained an inhibition of only 1.74%. While in the same type of study, against *Fusarium* sp., its behavior was very similar to that reported by other authors, with inhibition percentages of around 50%. This was not the case with *R. solani*, where the reported inhibitions were lower than those reported by the aforementioned author [40].

The variability of the *Trichoderma* species did not show a direct relationship with the plant species from which they were isolated, except *T. arundinaceum,* which was only present in one plant. The remaining species were found in at least two plant species, even in totally different plots. An explanation for this phenomenon could be that the environmental conditions affecting the root exudates that attract *Trichoderma* spp. are altered by environmental conditions. According to some authors, this phenomenon has a control on the type of strain that will be attracted to the plant [43,61].

The results obtained in our study agree with what has been expressed by other authors regarding the need to carefully select antagonistic strains according to the target pathogen and its interaction with the crop to achieve the maximum use of all its benefits in agriculture [36,62].

*Trichoderma* has a variety of direct mechanisms that allow it to control pathogenic organisms, such as competition for space, nutrients, production of cell wall-degrading enzymes, and antibiosis generated by various secondary metabolites [5]. All these mechanisms are involved in the dual-culture confrontation test. The first important factor in this test is the speed with which the little space delimited by the Petri dish is colonized; therefore, a point to highlight is that at least 50% of the isolates managed to colonize most of the dish quickly, even growing on the pathogen, thus proving to be excellent competitors.

The results from the dual-culture tests and the activity of volatile and soluble compounds demonstrated that the 20 selected isolates in this study significantly inhibited the growth of the pathogens responsible for chili wilt, the only exception being in the case of volatile compounds against *R. solani*. In the case of this pathogen, no strain managed to inhibit more than 10% of its growth. However, this behavior coincides with the work of Anees et al. [29], in which they characterized 16 isolates of *Trichoderma* from a soil producing sugar beet infected with *R. solani*, which did not show a noticeable inhibitory effect on the pathogen, obtaining inhibition percentages of less than 10%.

In this study, *P. capsici* and *Fusarium* sp. showed similar susceptibility to the mechanisms of action of the different strains of *Trichoderma.* The inhibition percentages were around 40 to 55% in the three tests. However, some strains had higher percentages than the average, even doubling them. These results are similar to those obtained by authors such as Mousumi Das et al. [63], who reported inhibition percentages from 32 to 49.8% in the case of *Phytophthora* and 35.02 to 44.3% for *Fusarium* spp.

## 5. Conclusions

The *Trichoderma* species identified by morphology and multilocus phylogenetic analyses with the ITS, RPB2, and TEF1 regions were *T. afroharzianum*, *T. lentiforme*, *T. rifaii*, *T. brevicompactum*, *T. arundinaceum*, and *T. longibrachiatum*. These species were mainly present in the rhizospheric soil of chili crops and wild plants.

*Trichoderma* strains demonstrated the ability to inhibit mycelial growth of the three phytopathogens that cause chili wilt (*R. solani*, *P. capsici*, and *Fusarium* sp.). The most antagonistic strains were *T. afroharzianum* 17RCS, *T. brevicompactum* 19RCS, *T. afroharzianum* 24RQS, *T. lentiforme* 63DPS, *T. longibrachiatum* 71JES, *T. rifaii* 77JCR, *T. afroharzianum* 87CCS, and *T. afroharzianum* 88CCS.

## Figures and Tables

**Table 1:** The list of *Trichoderma* isolates used in this study, and their origin and GenBank numbers.

Isolate	Municipality	Vegetal Species	Source	Species	ITS	TEF	RPB2
86CCS	Camargo	*Capsicum annuum*	Soil	*Trichoderma afroharzianum*	OR880629	PP263668	PP372571
87CCS	Camargo	*C. annuum*	Soil	*T. afroharzianum*	OR880630	PP263669	PP372572
88CCS	Camargo	*C. annuum*	Soil	*T. afroharzianum*	OR880631	PP263670	PP372573
57DPS	Delicias	*Picea* sp.	Soil	*T. lentiforme*	OR880621	PP273408	PP500715
58DPS	Delicias	*Picea* sp.	Soil	*T. lentiforme*	OR880622	PP273409	PP500716
63DPS	Delicias	*Picea* sp.	Soil	*T. lentiforme*	OR880623	PP273410	PP500717
70JES	Jiménez	*Salsola Kali*	Soil	*T. longibrachiatum*	OR880624	PP273411	PP526766
71JES	Jiménez	*S. Kali*	Soil	*T. longibrachiatum*	OR880625	PP273412	PP526767
73JCS	Jiménez	*C. annuum*	Soil	*T. rifaii*	OR880626	PP273413	PP500718
77JCR	Jiménez	*C. annuum*	Root	*T. rifaii*	OR880627	PP273414	PP500719
37MTS	Meoqui	*Solanum elaeagnifolium* Cav	Soil	*T. arundinaceum*	OR880618	PP263665	-
40MCS	Meoqui	*C. annuum*	Soil	*T. brevicompactum*	OR880619	PP263666	PP393118
42MZR	Meoqui	*Sorghum halepense* (L.) *Pers*	Root	*T. lentiforme*	OR880620	PP273407	PP500714
17RCS	Rosales	*C. annuum*	Soil	*T. afroharzianum*	OR880613	PP263660	PP372568
19RCS	Rosales	*C. annuum*	Soil	*T. brevicompactum*	OR880614	PP263661	PP372569
24RQS	Rosales	*Amaranthus* sp.	Soil	*T. afroharzianum*	OR880615	PP263662	PP372570
27RCS	Rosales	*C. annuum*	Soil	*T. brevicompactum*	OR880616	PP263663	PP393116
10SQS	Saucillo	*Amaranthus* sp.	Soil	*T. afroharzianum*	OR880612	PP263659	PP372567
33SCS	Saucillo	*C. annuum*	Soil	*T. afroharzianum*	OR880617	PP263664	-
85SCS	Saucillo	*C. annuum*	Soil	*T. brevicompactum*	OR880628	PP263667	PP393117

**Table 2:** Antagonistic effects of *Trichoderma* species against phytopathogenic microorganisms.

		*Rhizoctonia solani*			*Phytophthora capsici*			*Fusarium* sp.		
Species	Strain	Dual Culture	Non-VOC	VOC	Dual Culture	Non-VOC	VOC	Dual Culture	Non-VOC	VOC
*T. afroharzianum*	10SQS	39.367 ^abc^	19.747 ^bcd^	0 ^b^	74.2 ^abc^	47.157 ^de^	31.277 ^cd^	24.297 ^g^	45.713 ^fgh^	44.413 ^ab^
*T. afroharzianum*	17RCS	59.56 ^ab^	52.627 ^abcd^	5.297 ^ab^	48.953 ^def^	82.847 ^ab^	58.677 ^a^	62.147 ^abc^	80.9 ^b^	52.627 ^ab^
*T. brevicompactum*	19RCS	25.58 ^c^	42.237 ^abcd^	0 ^b^	60.523 ^abcde^	76.177 ^ab^	44.987 ^abc^	58.737 ^bc^	77.23 ^bc^	41.763 ^ab^
*T. afroharzianum*	24RQS	39.97 ^abc^	41.763 ^ab^	0 ^b^	45.463 ^def^	37.98 ^efg^	41.92 ^abcd^	61.647 ^abc^	12.223 ^k^	38.517 ^ab^
*T. brevicompactum*	27RCS	65.37 ^abc^	48.177 ^abc^	0 ^b^	43.03 ^ef^	34.143 ^efg^	30.613 ^cd^	54.92 ^cde^	48.57 ^bc^	48.177 ^ab^
*T. afroharzianum*	33SCS	61.283 ^ab^	18.75 ^cd^	2.6 ^ab^	56.217 ^bcde^	84.02 ^ab^	52.397 ^abc^	63.957 ^ab^	28.757 ^ij^	19.747 ^b^
*T. arundinaceum*	37MTS	41.86 ^abc^	56.223 ^bcd^	2.4 ^ab^	52.84 ^bcdef^	86.93 ^a^	3.853 ^e^	46.01 ^f^	75.653 ^bc^	22.017 ^b^
*T. brevicompactum*	40MCS	51.077 ^abc^	48.247 ^bcd^	0 ^b^	43.913 ^ef^	86.93 ^a^	20.733 ^de^	54.317 ^cdef^	78.183 ^bc^	48.247 ^ab^
*T. lentiforme*	42MZR	28.08 ^bc^	38.613 ^abcd^	1.25 ^ab^	44.66 ^ef^	28.613 ^g^	41.387 ^abcd^	49.537 ^def^	51.7 ^efg^	38.613 ^ab^
*T. lentiforme*	57DPS	28.593 ^bc^	60.273 ^a^	9.187 ^ab^	47.477 ^def^	26.383 ^g^	46.86 ^abc^	56.727 ^bcd^	22.72 ^jk^	58.64 ^a^
*T. lentiforme*	58DPS	29.027 ^bc^	58.64 ^a^	4.623 ^ab^	58.003 ^abcde^	70.54 ^bc^	51.757 ^abc^	58.633 ^bc^	35.51 ^hi^	55.423 ^a^
*T. lentiforme*	63DPS	34.543 ^abc^	39.16 ^a^	14.9 ^ab^	51.507 ^cdef^	58.943 ^cd^	54.46 ^ab^	60.34 ^abc^	42.337 ^gh^	60.273 ^a^
*T. longibrachiatum*	70JES	20.153 ^c^	46.83 ^abcd^	6.37 ^ab^	46.55 ^def^	58.913 ^cd^	39.067 ^abcd^	48.997 ^def^	71.933 ^cd^	46.83 ^ab^
*T. longibrachiatum*	71JES	48.313 ^abc^	35.04 ^abcd^	7.797 ^ab^	56.283 ^bcde^	36.077 ^efg^	45.857 ^abc^	47.193 ^ef^	60.963 ^de^	35.04 ^ab^
*T. rifaii*	73JES	18.69 ^c^	47.25 ^abcd^	0 ^b^	53.427 ^bcdef^	43.207 ^ef^	46.967 ^abc^	48.593 ^def^	100 ^a^	47.25 ^ab^
*T. rifaii*	77JCR	35.14 ^abc^	48.79 ^abc^	7.737 ^ab^	43.04 ^ef^	28.4 ^g^	55.523 ^ab^	49.597 ^def^	25.06 ^ij^	48.79 ^ab^
*T. brevicompactum*	85SQS	41.677 ^abc^	14.773 ^d^	0.933 ^b^	29.143 ^f^	86.93 ^a^	35.173 ^bcd^	68.977 ^a^	84.353 ^b^	46.997 ^ab^
*T. afroharzianum*	86CCS	29.373 ^bc^	37.523 ^abcd^	0 ^b^	76.587 ^ab^	31.553 ^fg^	47.35 ^abc^	54.117 ^cdef^	51.897 ^efg^	37.523 ^ab^
*T. afroharzianum*	87CCS	40.98 ^abc^	43.797 ^abcd^	0 ^b^	81.72 ^a^	32.153 ^fg^	55.42 ^ab^	55.12 ^cde^	57.54 ^def^	43.797 ^ab^
*T. afroharzianum*	88CCS	24.03 ^c^	36.057 ^abcd^	9.403 ^ab^	69.397 ^abcd^	29.103 ^fg^	56.333 ^ab^	56.63 ^bcd^	47.237 ^fgh^	36.057 ^ab^

Comparisons of means were made between each phytopathogenic fungus and bioassay technique/type. Equal-letter columns indicate that no statistical difference was found. The underlined figures correspond to the inhibition values of the images in Figure 4.

## Data Availability

The original contributions presented in the study are included in the article, further inquiries can be directed to the corresponding authors.

## Supplementary Materials

The following supporting information can be downloaded at: https://www.mdpi.com/article/10.3390/jof11030174/s1, Table S1. Dual-culture test of 88 isolates by municipality. Table S2. Non-volatile organic compounds test of 40 selected isolates by municipality. Table S3. Volatile organic compounds test of 40 selected isolates by municipality. Figure S1. Inhibition of phytopathogens mycelial of *R. solani*, *P. capsici* and *Fusarium* sp. growth by the 20 selected Trichoderma isolates. The blue diamond represents dual-confrontation data, the red squares represent the soluble compound activity test data and the green triangles correspond to the volatile compound activity test. The red line represents the 50% inhibition threshold. The blue letters correspond to the Tukey dual-confrontation mean separation test, the red letters to the soluble organic compound activity and the green letters to the volatile organic compound activity. Different letters within each color indicate statis-tical differences.
