# Supplementary material for: Trichoderma Species from Semiarid Regions and Their Antagonism Against the Microorganisms That Cause Pepper Wilt
Source: J Fungi (Basel). 2025 Feb 21;11(3):174. doi: 10.3390/jof11030174 (PMC11942951; doi:10.3390/jof11030174)
Supplement: Supplementary file 1 [file jof-11-00174-s001.zip › jof-3395232-supplementary.pdf]

Supplementary

Table S1.- Dual culture test of 88 isolates by municipality.

This evaluation was used as the first selection criterion. The best 40 isolates were selected to perform the following VOC and Non-VOC antagonism tests. The selection was made by taking the best isolates within each municipality.

	**Camargo**		
**Strain**	***Rhizoctonia solani***	***Phytophthora capsici***	***Fusarium* sp**
86CCS	29.373[a]	76.587[ab]	54.117[a]
87CCS	40.98[a]	81.72[ab]	55.12[a]
88CCS	24.03[a]	69.397[b]	56.63[a]
	**Delicias**		
**Strain**	***R. solani***	***P. capsici***	***Fusarium* sp**
51DPS	49.6[a]	47.7[ab]	48.09[d]
52DPS	21.877[ab]	50.92[ab]	52.31[d]
53DPS	29.8[ab]	51.797[ab]	52.933[bcd]
54DPS	36.217[ab]	51.81[ab]	53.313[bcd]
55DPS	45.477[ab]	56.633[ab]	51.203[cd]
56DPS	17.313[b]	64.82[ab]	45.983[de]
57DPS	28.593[ab]	47.477[ab]	56.727[abc]
58DPS	29.027[ab]	58.003[ab]	58.633[abc]
59DPS	38.587[ab]	60.35[ab]	63.153[a]
60DPS	28.767[ab]	66.51[ab]	52.713[a]
61DPS	43.62[ab]	62.517[ab]	38.957[cd]
62DPS	31.653[ab]	54.91[ab]	61.043[a]
63DPS	34.543[ab]	51.507[ab]	60.34[abc]
64DSP	43.537[ab]	66.513[ab]	51.707[cb]
65DPS	35.4[ab]	59.04[ab]	62.15[a]
	**Jimenez**		
**Strain**	***R. solani***	***P. capsici***	***Fusarium***
66JES	31.827[ab]	55.29[abcd]	43.977[bc]
67JES	29.637[ab]	57.313[abc]	54.32[ab]
68JCS	38.74[ab]	64.307[ab]	55.327[a]
69JCS	42.737[ab]	54.33[abcde]	45.983[abc]

70JES	20.153^{ab}	46.55^{abcde}	48.997^{abc}
71JES	48.313^{ab}	56.283^{abc}	47.193^{abc}
72JES	34.527^{ab}	54.657^{abcde}	46.887^{abc}
73JES	18.69^{ab}	53.427^{abcde}	48.593^{abc}
74JCS	31.707^{ab}	50.28^{abcde}	44.277^{bc}
75JCS	5.537^{a}	51.29^{abcde}	53.717^{abc}
76JES	50.197^{ab}	64.237^{ab}	53.367^{abc}
77JCR	35.14^{ab}	43.04^{bcde}	49.597^{abc}
78JCS	17.483^{b}	29.627e	42.72c
79JCS	34.623^{ab}	64.21^{ab}	48.793^{abc}
80JCS	28.593^{ab}	30.34de	49.8^{abc}
81JCS	31.56^{ab}	32.313cde	51.007^{abc}
82JCS	23.6^{ab}	64.287^{ab}	52.61^{abc}
83JCS	32.213^{ab}	70.2^{a}	53.917^{abc}
84JCS	17.57^{b}	32.867^{cde}	44.76^{bc}
**Meoqui**			
**Strain**	***R. solani***	***P. capsici***	***Fusarium* sp**
36MTS	15.507^{e}	56.453^{abc}	40.063^{bc}
37MTS	41.86^{abcd}	52.84^{abc}	46.01^{ab}
38MTS	54.93^{a}	48.173^{abc}	44.76^{abc}
39MTS	49.87^{abc}	57.837^{ab}	42.973^{bc}
40MCS	51.077^{ab}	43.913^{abc}	54.317^{ab}
41MZS	35.287^{abcde}	60.833^{a}	60.14^{a}
42MZR	28.08^{bcde}	44.66^{abc}	49.537^{ab}
43MZR	30.493^{bcde}	33.333^{bc}	46.85^{ab}
44MZR	30.837^{bcde}	61.24^{a}	41.313^{bc}
45MZR	27.993^{bcde}	56.453^{abc}	51.807^{ab}
46MZR	26.44^{cde}	55.837^{abc}	53.513^{ab}
47MZR	35.23^{abcde}	43.697^{abc}	43.66^{bc}
48MZS	33.763^{abcde}	58.91^{ab}	41.627^{bc}
49MZS	23.43^{de}	63.36^{a}	47.79^{ab}
50MZS	18.95^{de}	31.037^{c}	30.04^{c}
**Rosales**			

Strain	*R. solani*	*P. capsici*	*Fusarium* sp
17RCS	59.56^{a}	48.953^{abc}	62.147^{a}
18RCS	31.78^{ab}	40.57^{abc}	50.99^{abcd}
19RCS	25.58^{ab}	60.523^{ab}	58.737^{ab}
20RCS	51.59^{ab}	59.523^{ab}	50.153^{bcd}
21RCS	43.47^{ab}	32.367	55.167^{abc}
23RCS	37.23^{ab}	58.8^{abc}	47.263^{cd}
24RQS	39.97^{ab}	45.463^{abc}	61.647^{a}
25RCS	42.02^{ab}	55.247^{abc}	53.917^{abc}
26RCS	38.54^{ab}	55.99^{abc}	45.383^{cd}
27RCS	65.37^{a}	43.03^{abc}	54.92^{abc}
28RCS	39.03^{ab}	63.363^{a}	49.277^{bcd}
29RCS	17.22^{b}	35.903^{bc}	41.443^{d}
35RQS	54.587^{ab}	49.153^{ab}	64.46^{a}

Saucillo

Strain	*R. solani*	*P. capsici*	*Fusarium* sp
1SCS	32.043^{bcdefgh}	58.68^{abc}	64.357^{abc}
2SQS	26.357^{defghi}	34.31^{fg}	44.578^{abc}
3SCS	17.227^{fghi}	37.967^{defg}	62.75^{fg}
4SCS	5.943^{i}	58.727^{abc}	49.733^{abcd}
5SCS	15.16^{hi}	46.707^{bcdefg}	54.02^{defg}
6SQS	16.967^{fghi}	54.033^{bcde}	38.957^{cdef}
7SQS	16.45^{fghi}	39.877^{cdefg}	45.853^{g}
8SCS	24.117^{defghi}	58.333^{abc}	55.997^{fg}
9SQS	39.533^{abcdef}	46.84^{bcdefg}	63.95^{7bcdef}
10SQS	39.367^{abcdefg}	74.2^{a}	24.297^{h}
11SQS	44.277^{abcd}	35.807^{efg}	38.577^{efg}
12SQS	56.733^{a}	49.17^{bcdef}	60.343^{g}
13SQS	45.223^{abcd}	46.81^{bcdefg}	60.74^{abcde}
14SQS	49.873^{abc}	49.17^{bcdef}	65.867^{abcde}
15SQS	46.76^{abcd}	57.34^{abcd}	65.263^{abc}
16SCS	42.393^{abcd}	65.82^{ab}	43.447^{abc}
22SQS	18.603^{efghi}	36.113^{efg}	62.853^{fg}

30SQS	44.96^{abcd}	57.56^{abcd}	54.92^{abcd}
31SCS	30.923^{cdefgh}	51.767^{bcdef}	69.98^{cdef}
32SCS	15.937^{ghi}	39.773^{defg}	68.977^{a}
33SCS	61.283^{a}	56.217^{abcd}	63.957^{abc}
34SCS	43.08^{abcd}	56.683^{abcd}	37.453^{fg}
85SCS	41.677^{abcde}	29.143^{g}	68.977^{ab}

Comparisons of means were made between each phytopathogenic fungus and municipality. Equal letters columns indicate that no statistical difference was found.

Table S2.- Non-volatile organic compounds test of 40 selected isolates by municipality.

The strains that presented the best results in this test and the VOCs test (S3) were selected as the 20 best isolates, on which phylogenetic identification at species level would be carried out. The selection was made by taking the best isolates within each municipality.

**Camargo**			
**Strain**	***Rhizoctonia***	***Phytophthora***	***Fusarium***
86CCS	43.797^{a}	31.553^{a}	51.897^{a}
87CCS	37.523^{a}	32.153^{a}	57.54^{a}
88CCS	36.057^{a}	29.103^{a}	47.237^{a}
**Delicias**			
**Strain**	***Rhizoctonia***	***Phytophthora***	***Fusarium***
57DPS	60.273^{a}	70.54^{a}	22.72^{bc}
58DPS	58.64^{a}	70.187^{a}	35.51^{ab}
59DPS	55.423^{ab}	58.943^{a}	16.067^{c}
62DPS	50.757^{ab}	58.833^{a}	21.17^{c}
63DPS	39.16^{ab}	31.23^{b}	42.337^{a}
65DPS	26.323^{bc}	26.383^{b}	23.808^{bc}
**Jiménez**			
**Strain**	***Rhizoctonia***	***Phytophthora***	***Fusarium***
67JES	32.76^{a}	21.397^{de}	25.977^{c}
68JCS	36.27^{a}	13.937^{e}	13.65^{c}
69JCS	47.8^{a}	53.037^{ab}	18.973^{c}
70JES	46.83^{a}	58.913^{a}	71.933^{b}
71JES	35.04^{a}	36.077^{bcd}	60.963^{b}
73JES	47.25^{a}	43.207^{ab}	100^{a}
75JCS	45.6^{a}	57.39^{a}	27.007^{c}
77JCR	48.79^{a}	28.4^{cde}	25.06^{c}
**MEOQUI**			
**Strain**	***Rhizoctonia***	***Phytophthora***	***Fusarium***
37MTS	56.223^{a}	86.93^{a}	75.653^{a}
38MTS	38.613^{abc}	54.397^{bc}	74.863^{a}
40MCS	48.247^{ab}	86.93^{a}	78.183^{a}
41MZS	33.1^{bc}	52.71^{bc}	21.71^{c}
42MZR	29.473^{bc}	28.613^{c}	51.7^{bc}
47MZR	22.017^{c}	41.903^{bc}	22.99^{c}

48MZS	24.543^{c}	28.86^{c}	34.303^{bc}
**Rosales**			
**Strain**	***Rhizoctonia***	***Phytophthora***	***Fusarium***
17RCS	52.627^{a}	82.847^{b}	80.9^{a}
18RCS	28.287^{a}	53.257^{b}	79.693^{a}
19RCS	42.237^{a}	76.177^{a}	77.23^{a}
24RQS	41.763^{a}	37.98^{b}	67.573^{b}
25RCS	38.517^{a}	79.58^{a}	48.57^{c}
27RCS	48.177^{a}	34.143^{a}	12.223^{d}
**Saucillo**			
**Strain**	***Rhizoctonia***	***Phytophthora***	***Fusarium***
9SQS	23.96^{a}	66.54^{cd}	38.37^{bcd}
10SQS	19.747^{a}	47.157^{ef}	45.713^{b}
12SQS	19.173^{a}	60.143^{cde}	15.473^{gh}
13SQS	15.08^{a}	31.937^{f}	10.327^{h}
14SQS	16.45^{a}	52.03^{de}	16.88^{fgh}
15SQS	32.127^{a}	69.753^{bc}	42.163^{bc}
16SCS	36.34^{a}	67.247^{cd}	29.917^{cde}
30SQS	35.417^{a}	66.13^{cd}	23.633^{efg}
33SCS	18.75^{a}	84.02^{ab}	28.757^{def}
34SCS	14.533^{a}	45.03^{ef}	21.07^{efgh}
85SCS	14.773^{a}	86.93^{a}	84.353^{a}

Table S3.- Volatile organic compounds test of 40 selected isolates by municipality.

**Camargo**			
**Strain**	***Rhizoctonia***	***Phytophthora***	***Fusarium***
86CCS	0^{a}	47.35^{a}	37.523^{a}
87CCS	0^{a}	55.42^{a}	43.797^{a}
88CCS	9.403^{a}	56.333^{a}	36.057^{a}
**Delicias**			
**Strain**	***Rhizoctonia***	***Phytophthora***	***Fusarium***
57DPS	9.187^{a}	46.86^{a}	58.64^{a}
58DPS	4.623^{a}	51.757^{a}	55.423^{ab}
59DPS	0^{a}	45.84^{a}	39.16^{ab}
62DPS	0^{a}	54.227^{a}	50.757^{ab}
63DPS	14.9^{a}	54.46^{a}	60.273^{a}
65DPS	0^{a}	58.167^{a}	26.313^{b}
**Jiménez**			
**Strain**	***Rhizoctonia***	***Phytophthora***	***Fusarium***
67JES	3.057^{ab}	29.803^{de}	32.76^{a}
68JCS	0^{b}	22.073^{e}	36.27^{a}
69JCS	0^{b}	26.273^{e}	47.8^{a}
70JES	6.37^{ab}	39.067^{bcde}	46.86^{a}
71JES	7.797^{ab}	45.857^{abcd}	35.04^{a}
73JES	0^{b}	46.967^{abc}	47.25^{a}
75JCS	0^{b}	28.787^{de}	45.6^{a}
77JCR	7.737^{ab}	55.523^{ab}	48.79^{a}
**MEOQUI**			
**Strain**	***Rhizoctonia***	***Phytophthora***	***Fusarium***
37MTS	2.4^{a}	3.853^{b}	22.017^{b}
38MTS	0^{a}	13.133^{ab}	21.543^{b}
40MCS	0^{a}	20.733^{ab}	48.247^{a}
41MZS	0^{a}	38.45^{a}	56.223^{a}
42MZR	1.25^{a}	41.387^{a}	38.613^{ab}
47MZR	4.187^{a}	33.873^{ab}	50.283^{a}
48MZS	0.813^{a}	27.487^{abc}	46.213^{a}
**Rosales**			
**Strain**	***Rhizoctonia***	***Phytophthora***	***Fusarium***
17RCS	5.297^{a}	32.597^{b}	38.517^{a}
18RCS	0^{a}	58.677^{a}	42.237^{a}

19RCS	0^{a}	56.313^{a}	41.763^{a}
24RQS	0^{a}	41.92^{ab}	52.627^{a}
25RCS	2.6^{a}	30.613^{b}	28.287^{a}
27RCS	0^{a}	44.987^{ab}	48.177^{a}
**Saucillo**			
**Strain**	***Rhizoctonia***	***Phytophthora***	***Fusarium***
9SQS	0^{a}	32.657^{b}	48.343^{a}
10SQS	0^{a}	52.397^{a}	19.747^{b}
12SQS	3.69^{a}	34.593^{b}	50.947^{a}
13SQS	0^{a}	31.593^{b}	53.693^{a}
14SQS	0^{a}	31.147^{b}	49.193^{a}
15SQS	3.89^{a}	44.05^{ab}	32.127^{ab}
16SCS	4.663^{a}	42.26^{ab}	36.343^{ab}
30SQS	0^{a}	51.82^{a}	35.417^{ab}
33SCS	2.6^{a}	35.173^{b}	46.997^{a}
34SCS	0.933^{a}	33.233^{b}	45.62^{a}
85SCS	0^{a}	31.277^{b}	44.413^{a}

Comparisons of means were made between each phytopathogenic fungus and municipality. Equal letters columns indicate that no statistical difference was found.

Figure S1.- Inhibition of phytopathogens mycelial growth by selected *Trichoderma* isolates. The blue diamonds represent the dual confrontation data, the red squares represent the soluble compound activity test data and the green triangles correspond to the volatile compound activity test. The red line represents the 50% inhibition threshold. The blue letters correspond to the Tukey dual confrontation mean separation test, the red letters to the soluble compound activity and the green letters to the volatile compound activity. Different letters within each color indicate statistical difference according to Tukey's test ($p \leq 0.05$).

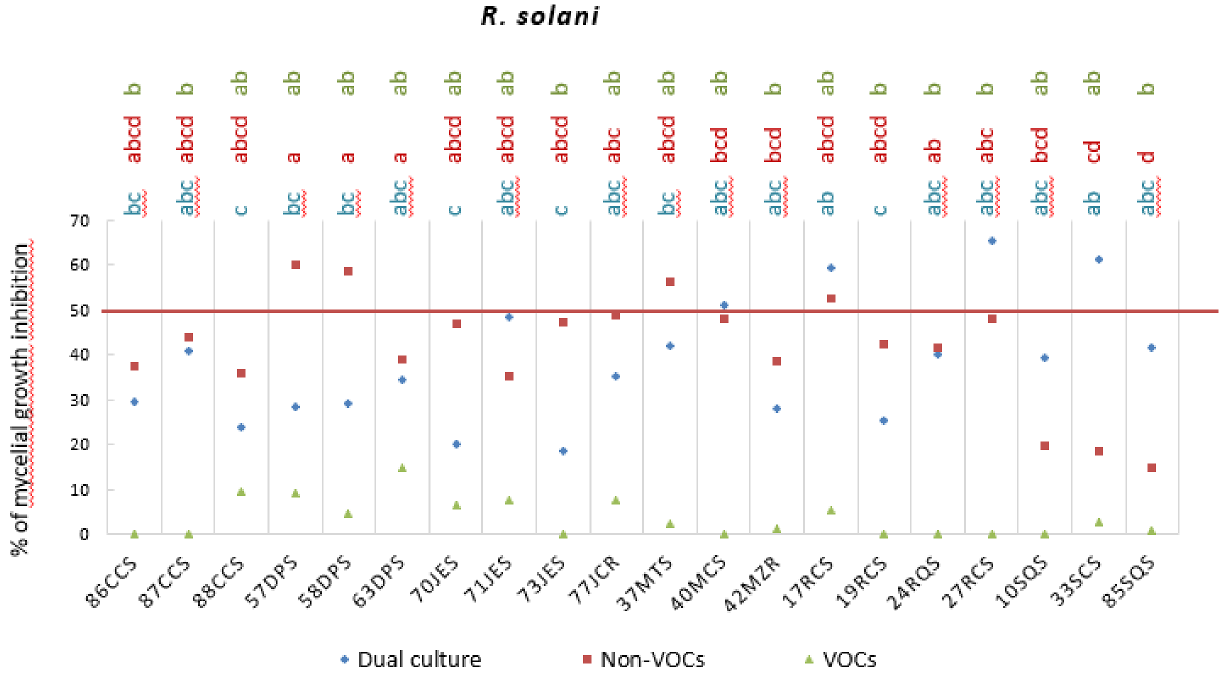

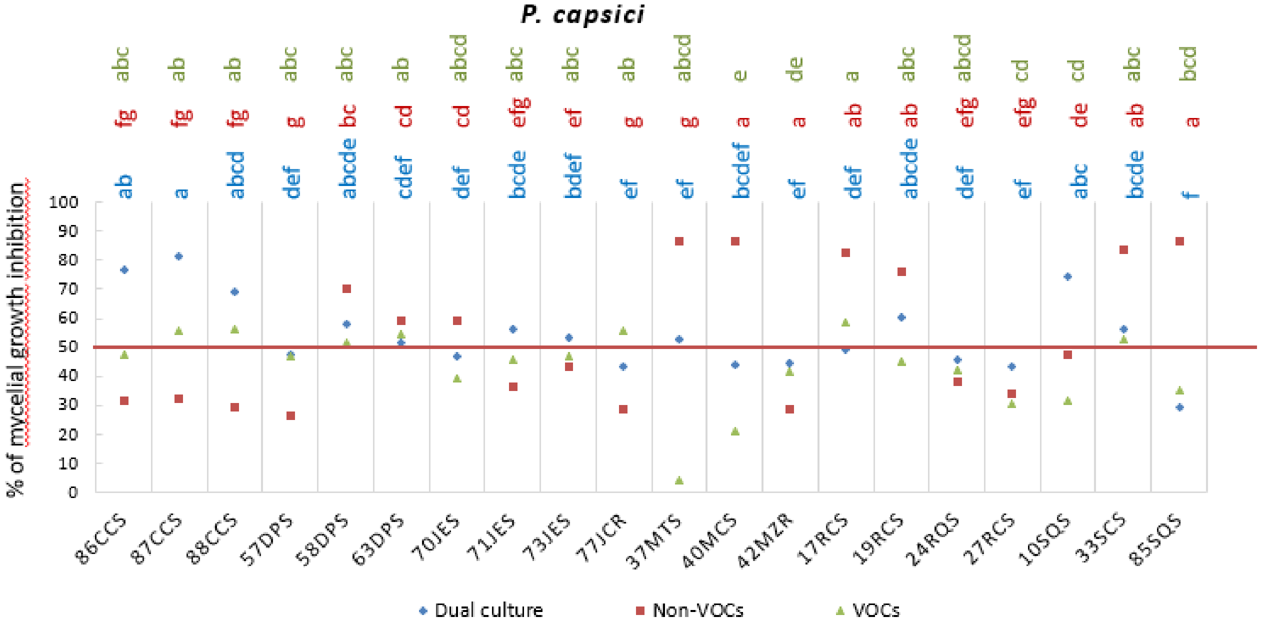

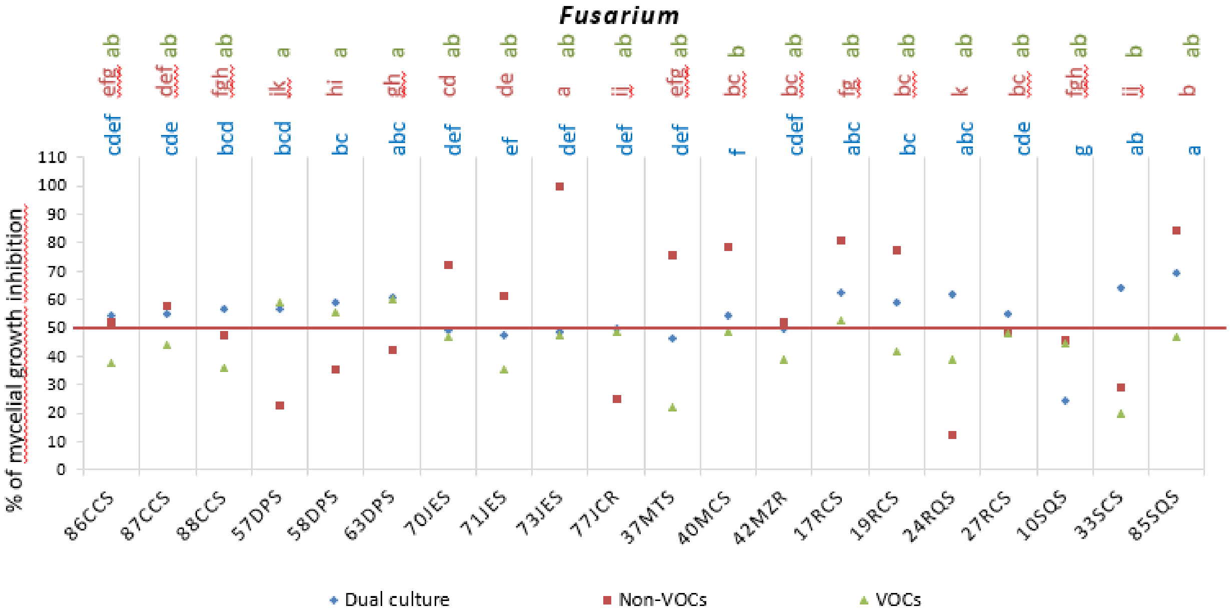

Fusarium
% of mycelial growth inhibition
86CCS
87CCS
88CCS
57DPS
58DPS
63DPS
70JES
71JES
73JES
77JCR
37MTS
40MCS
42MZR
17RCS
19RCS
24RQS
27RCS
10SQS
33SCS
85SQS
Dual culture
Non-VOCs
VOCs
